# Analysis of the coding sequences of clownfish reveals molecular convergence in the evolution of lifespan

**DOI:** 10.1186/s12862-019-1409-0

**Published:** 2019-04-11

**Authors:** Arne Sahm, Pedro Almaida-Pagán, Martin Bens, Mirko Mutalipassi, Alejandro Lucas-Sánchez, Jorge de Costa Ruiz, Matthias Görlach, Alessandro Cellerino

**Affiliations:** 1 Leibniz Institute on Aging, Fritz Lipmann Institute, Jena, Germany; 2 Departamento de Fisiologia, Universidad de Murcia, Murcia, Spain; 3 Stazione Zoologica Anton Dohrn, Naples, Italy; 4 Bio@SNS, Scuola Normale Superiore, Pisa, Italy

**Keywords:** Amphiprion, Positive selection, Evolution of lifespan, Life-history trait, Mito-nuclear balance

## Abstract

**Background:**

Standard evolutionary theories of aging postulate that reduced extrinsic mortality leads to evolution of longevity. Clownfishes of the genus Amphiprion live in a symbiotic relationship with sea anemones that provide protection from predators. We performed a survey and identified at least two species with a lifespan of over 20 years. Given their small size and ease of captive reproduction, clownfish lend themselves as experimental models of exceptional longevity. To identify genetic correlates of exceptional longevity, we sequenced the transcriptomes of *Amphiprion percula* and *A. clarkii* and performed a scan for positively-selected genes (PSGs).

**Results:**

The PSGs that we identified in the last common clownfish ancestor were compared with PSGs detected in long-lived mole rats and short-lived killifishes revealing convergent evolution in processes such as mitochondrial biogenesis. Among individual genes, the Mitochondrial Transcription Termination Factor 1 (*MTERF1*), was positively-selected in all three clades, whereas the Glutathione S-Transferase Kappa 1 (*GSTK1*) was under positive selection in two independent clades. For the latter, homology modelling strongly suggested that positive selection targeted enzymatically important residues.

**Conclusions:**

These results indicate that specific pathways were recruited in independent lineages evolving an exceptionally extended or shortened lifespan and point to mito-nuclear balance as a key factor.

**Electronic supplementary material:**

The online version of this article (10.1186/s12862-019-1409-0) contains supplementary material, which is available to authorized users.

## Background

The lifespan of vertebrate species spans more than two orders of magnitude, from a few months for annual killifishes [1] to several centuries for the Greenland shark [2]. Understanding the genetic architecture underlying these differences is a major challenge that may deliver new insights into the mechanisms controlling evolution of lifespan and human longevity.

Next-generation sequencing technology can provide genome-scale sequence information for a large number of species and has revolutionized evolutionary genomics. A particularly useful approach to identify the genetic architecture of evolutionary novelties is the analysis of positive selection. A frequently used method for detecting positive selection relies on the comparison of the sequence of protein-coding genes in related clades where one of the clades evolved the trait of interest, in this specific case exceptional lifespan. To date, several different mammalian taxa/clades were analysed with this approach with the purpose of identifying sequence changes associated to evolution of longevity: the elephant, the bowhead whale, bats and mole-rats [3–8]. In addition to the analysis of positive selection, other phylogeny-based methods have been used to investigate genetic changes related to the evolution of longevity, e.g. [9–12]. The different analyses delivered interesting candidate genes and pathways that underwent accelerated molecular evolution in coincidence with evolution of exceptional lifespan. A major drawback of all these approaches – including the analysis of positive selection – is that long-lived mammals are difficult or impossible to be kept in captivity and manipulated experimentally. This creates the need for a long-lived vertebrate that is small in size, easily adaptable to captive life, can be bred in large numbers and therefore could become a convenient experimental model organism.

The standard evolutionary theories of aging – namely the Mutation Accumulation [13], Antagonistic Pleiotropy [14] and Disposable Soma theory [15] – predict that low extrinsic mortality conditions lead to the evolution of slow senescence and increased lifespan. Some examples that confirm these theories are the exceptional longevity of vertebrate species under low predation risk since they are chemically protected [16, 17], adapted to an arboreal life [18] or found in protected environments such as caves [19], respectively. On the other hand, annual fishes of the genus *Nothobranchius* provide an example of how increased extrinsic mortality conditions lead to the evolution of accelerated senescence and a short lifespan [20–22]. Analysis of positive selection in annual killifishes revealed a potential link between the evolution of genes governing mitochondrial biogenesis and the evolution of lifespan [23].

All clownfish species (genus *Amphiprion*) evolved a specific adaptation that allows them to live in symbiosis with sea anemones. Symbiosis evolved in the last common ancestor of the clownfish and clownfish represent a monophyletic group in the Pomacentridae family (damselfishes) [24]. In the Indo-Pacific Ocean, clownfishes are found in association with one or more sea anemone species and a large variation in host usage exists [25–27]. Fish that feel threatened by predators immediately seek protection by the anemone’s tentacles; without that symbiosis, fish are readily attacked by predators [28–30]. Therefore, clownfishes are protected from predators through the presence of anemones [31]. Hence, the overall mortality rate of the clownfish is low as compared to other coral reef fishes or other tropical species of Pomacentridae of the same size [28, 31–34].

All clownfishes are born as males and develop, through protandrous hermaphroditism, into females: in a colony, only the dominant pair contributes to the reproduction of the colony [35]. Other individuals of the colony are non-breeding males. Studies in the wild have shown that natural mortality of adult clownfishes can be very low: during the period 2011–2013, the average biannual mortality rate per capita varied, depending on the study site, between 0.18 and 0.49 for juveniles, 0.09 and 0.44 for males, and 0.19 and 0.55 for females [36]. Predatory pressure differs in different stages of adulthood and is increased for non-breeding males [28].

These fishes are small in size (less than 10 cm for the smallest species) and the closely-related species *A. percula* and *A. ocellaris* are popular and hardy aquarium fishes, are bred in large numbers for the aquarium trade [37] and are subject to selective breeding to fix specific pigmentation patterns so that a number of different captive strains are available. For these reasons, the clownfish could become the first experimental model for long-lived vertebrates.

In order to identify the genetic basis of adaptations linked to the clownfishes’ exceptional lifespan, we performed a positive selection analysis. This analysis requires the identification of the closest related taxon that does not possess the trait of interest in order to exclude events of positive selection that predate the evolution of this trait [38].

**Fig. 1:**
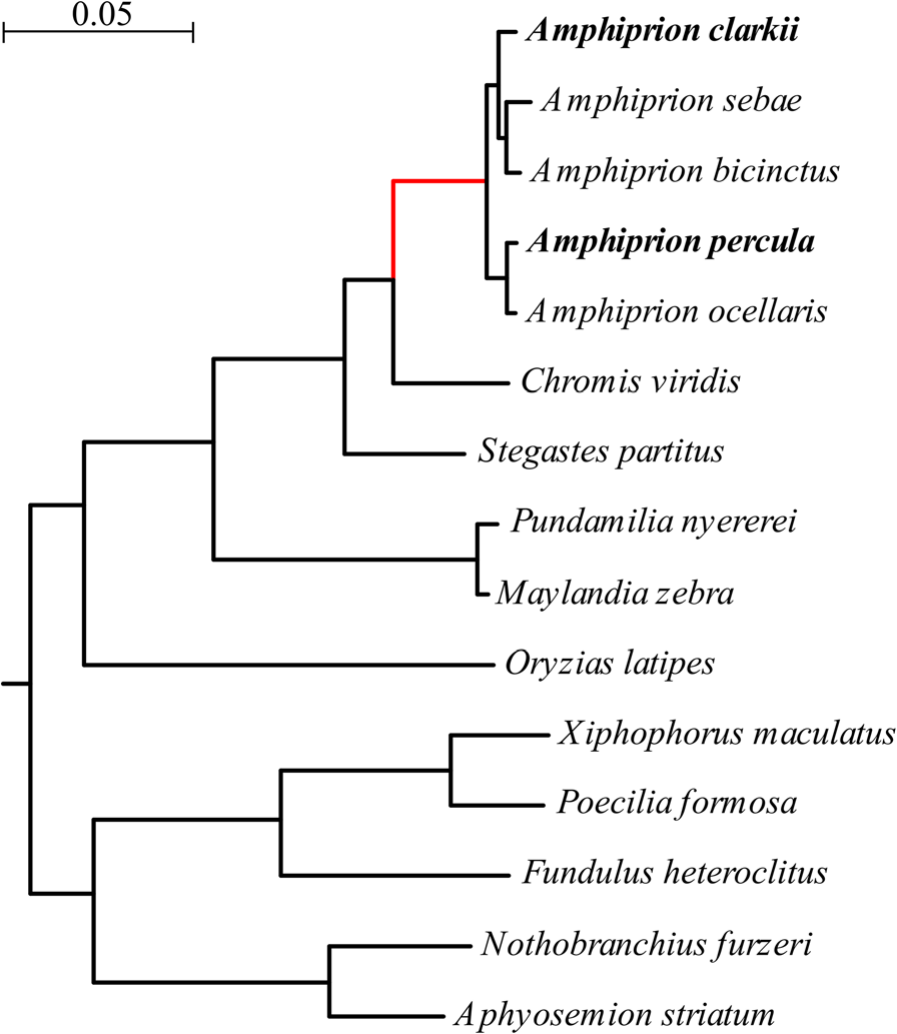
Nucleotide-based phylogeny of the analyzed fish species. We searched for positively selected genes on the last common ancestor of the clownfishes (*Amphiprion*, red). The two species *A. clarkii* and *A. percula* depicted in bold are those that were sequenced in this study. The phylogenetic tree was derived as part of the positive selection analysis with the PosiGene pipeline [79]. Briefly, during this process 8215 genes were concatenated and the resulting concatenated alignment split in 404 fragments each of which had a length of 15,000 nucleotides. From each fragment, a phylogeny was calculated via maximum likelihood and, from all resulting 404 trees, a consensus tree was determined using the Phylip package [83]. The scale bar represents 0.05 substitutions per site

Other species of damselfish evolved an inter-specific mutualistic relationship with branching corals [39, 40]. In this case, corals are used by fishes as shelter that can provide protection from predators and a safe area for egg laying [41, 42]. Among the family Pomacentridae, *Chromis viridis* shows an interesting relationship with a wide range of scleractinians [43, 44]. Despite the presence of a favourable microhabitat, *C. viridis* are predated by a wide range of generalist predator species. Hixon and Carr [45] suggested there is a clear relationship among transient and benthic predators and damselfish mortality: damselfishes that search for protection in the shelter from transient predators are susceptible to attack by resident benthic predators and vice versa. In the presence of both groups of predators, mortality increases dramatically due to the lack of available refuge that expose *Chromis* to intense predation [45]. Therefore, *Chromis viridis* represents a well-suited outgroup for our analysis because it shares with clownfish several general traits linked to benthic life and symbiosis with corals but it is subject to much higher predation rates (Fig. 1).

There are no reliable estimates of *C. viridis* lifespan in captivity. This is mainly due to the fact that these animals build large schools and it is not possible to identify them individually, as opposed to clownfishes that are normally kept as pairs and, if multiple pairs are maintained in the aquarium, each pair associates to a different symbiont making their distinction easy. For the same reason, the presence of a specific clownfish on a specific anemone can be assessed in multiple years to obtain the rates of per annum mortality [36], which is not possible for *C. viridis*. However, it is certain that tropical Chromis fishes undergo severe predation in the post-settlement phase [45]. In one experiment where the total number of the population was assessed, a decrease in the population size of almost 80–90% in one year was documented. Notably, this not only affects juvenile fish, but also adults, indicating high adult mortality. This, combined with a very rapid growth (80% of maximum size reached within the first year) clearly indicates that these animals are short-lived in the wild [46]. Indeed, they are considered a priori a model for short-lived reef inhabitants [46]. Mortality data and age structure are available for the temperate species *Chromis chromis* (that, due to lower temperatures, may be longer-lived than tropical species). In this case, the natural adult annual mortality was estimated to be 0.72 and only < 7% of the individuals reached ages higher than 7 years [47].

## Results

### Lifespan-data confirms that clownfish evolved exceptional long lifespans

Several anecdotic reports are present in the hobbyist literature on the exceptional lifespan of the clownfish. In order to obtain independent data on the captive lifespan of clownfish species, in 2016 we distributed a questionnaire to researchers working with clownfishes and to public aquaria across Europe (Table 1 and Additional file 1: Table S1) resulting in lifespan data for, in total, 114 individuals. Additionally, we surveyed existing literature. For six different species, at least one individual was reported to have lived more than 10 years and for two different species, *A. melanopus* and *A. ocellaris*, we obtained records of animals alive and actively spawning at an age of over 20 years, confirming hobbysts’ reports. This indicates that even the longest-lived individuals observed in captivity did not show reproductive senescence and were not approaching the limit of their lifespan.

**Table 1:** Results of the clownfish survey. The longest-lived individual for each species is indicated

Species	Maximum length (mm)^c^	oldest animal (years)	status at census	size of group
*A. akydinos*	90	13	dead	1
*A. clarkii (wild)* ^a^	150	12	alive	n.a.
*A. clarkii (privately owned)*	150	16	alive	2/0 dead
*A. clarkii*	150	9	alive	2/0 dead
*A. frenatus* ^b^	140	18	dead	n.a.
*A. melanopus*	120	21	alive	2/0 dead
*A. ocellaris (privately owned)*	110	22	alive	2/0 dead
*A. ocellaris*	110	17	alive	2/0 dead
*A. perideraion* ^b^	100	18	alive	n.a.

^a^ Moyer, 1986 [92]

^b^ Fautin and Allen, 1992 [25]

^c^ Maximum size from Fishbase [93]

More systematic data could be obtained for the species *A. ocellaris* (the most common species in the aquarium trade). The oldest cohort for which a record was available comprised 27 fish born in 2008 of which 25 were still alive in 2016.

We conclude that there is solid evidence that at least the species *A. ocellaris* and *A. melanopus* can live in captivity for more than two decades, making them the first teleost model of exceptional longevity.

### Genes that were positively selected on the ancestral clownfish branch are enriched for aging-relevant functions

In order to perform genome-wide scans for positive selection, we obtained the transcriptomes of the species *A. clarkii* and *A. percula* based on our sequencing using methods previously described for the killifishes [23]. Furthermore, we assembled clownfish transcriptomes from public read data of *A. bicinctus*, *A. ocellaris* and *A. sebae*. As the closest-related non-symbiotic species, we additionally sequenced the transcriptome of *Chromis viridis*, a closely-related Pomacentrid of 10 cm maximum size, i.e. comparable to that of the smallest clownfish species (Table 1), which is very abundant in coral reefs and lacks adaptations for symbiosis with sea anemones. Although data on *C. viridis* mortality in the wild are not available, the higher predation pressure on this species (see Background) makes it a suitable outgroup for our analysis. More distant outgroups were a selection of species from the series Ovalentaria, whose genomes are available in GenBank (see also [23]). We analysed positive selection on the branch leading to the last common ancestor (LCA) of all clownfish species (Fig. 1).

A total of 157 positively selected genes (PSGs) of 14,214 analyzed genes were identified in the LCA of the clownfishes (Additional file 1: Table S2). We tested for overrepresentation of gene ontology (GO, FDR < 0.1) and observed 19 biological processes enriched for PSGs (Table 2, Additional file 1: Table S3). A majority of these processes are of particular interest for aging research: altogether nine enriched processes are linked to the metabolism of xenobiotics, detoxification or glutathione metabolism, respectively. Interestingly, these processes were shown to be strongly up-regulated in experimental conditions favoring longevity such as dietary restriction and inhibition of the somatotropic axis making the animals more resistant to toxins [48–51]. Furthermore, experimental manipulation of mitochondrial translation, another enriched process, is known to increase lifespan in *C. elegans* [52] and variations in the expression of these genes is associated to lifespan variation in killifish [53] and mouse [52]. We also tested formally the relationship between the results of the positive selection analysis on the LCA of the clownfishes and aging. For this, we checked whether processes whose gene expression was shown to be associated with maximum longevity in an analysis across 33 diverse mammal species [12], were also enriched during the positive selection analysis, which was the case (*p* = 0.06).

**Table 2:** Biological gene ontology processes enriched for positively selected genes (FDR < 0.1)

GOBPID^a^	Term	FDR^b^
GO:1901685	glutathione derivative metabolic process	0.021
GO:1901687	glutathione derivative biosynthetic process	0.021
GO:0006805	xenobiotic metabolic process	0.084
GO:0032543	mitochondrial translation	0.084
GO:0071466	cellular response to xenobiotic stimulus	0.084
GO:0009410	response to xenobiotic stimulus	0.084
GO:0042178	xenobiotic catabolic process	0.084
GO:0007157	heterophilic cell-cell adhesion via plasma membrane cell adhesion molecules	0.084
GO:0050900	leukocyte migration	0.084
GO:0045321	leukocyte activation	0.084
GO:0007155	cell adhesion	0.084
GO:0022610	biological adhesion	0.084
GO:0048870	cell motility	0.084
GO:0051674	localization of cell	0.084
GO:1990748	cellular detoxification	0.084
GO:0055081	anion homeostasis	0.084
GO:0007229	integrin-mediated signaling pathway	0.086
GO:0016477	cell migration	0.086
GO:0098754	detoxification	0.086

^a^ GOBPID – gene ontology biological process ID

^b^ FDR – false discovery rate (adjusted *p*-value for multiple testing, see methods for an explanation of same values in many rows)

**Fig. 2:**
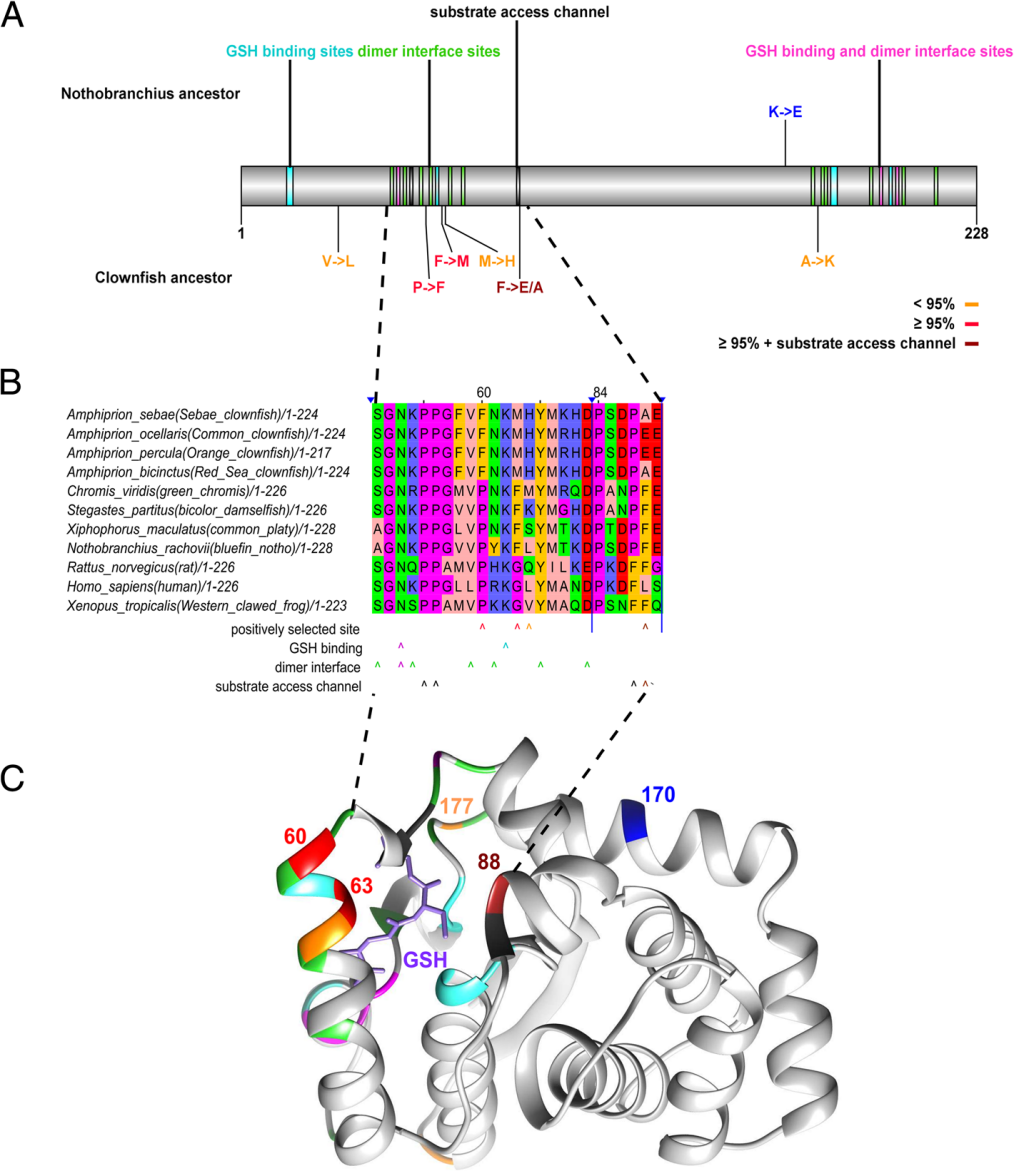
**a** Linear depiction of GSTK1 with color coded known functional domains/sites (dimer interface – green, GSH binding – turquoise, sites that serve as both dimer interface and GSH binding – violet, substrate access channel – black) and positively selected sites (in the last common ancestor of the clownfishes with a predicted probability ≥95% – red, in the last common ancestor of the clownfishes with a predicted probability < 95% – orange, in the last common ancestor of *Nothobranchius pienaari* and *Nothobranchius rachovii* – blue. **b** Alignment of GSTK1 orthologs across a wide phylogenetic range of species. Depicted are two protein regions (51–69, 84–89) that contain positively selected sites and functionally relevant sites in close proximity. The color code for positively selected and functionally relevant sites is the same as in panel A. **c** Clownfish GSTK1 model showing one subunit of the modelled dimer (for an overview see Additional file 3: Figure S1). Selected positions are color coded according to function depicted in the overview scheme at the top. The numbered and colored residue positions (60, 68, 88, 170 and 177) are discussed in detail in the text. Also shown is the GSH substrate (glutathione, light purple) as positioned in the template structure (PDB 1R4W) of the rat GSTK1

The positive selection analysis provides not only candidate genes but also candidate amino acids for follow-up studies. To exemplify this, we performed protein homology modeling for GSTK1 starting from the publicly available structures of the human dimeric apoenzyme (PDB 3RPP, [54]) and the rat dimeric enzyme with the bound GSH substrate (PDB 1R4W; [55]). The latter was used to assess on a structural basis the relationship of the six positively selected sites in the clownfish with those that are known to be involved in the enzyme’s function [55]. Interestingly, also the LCA of Nothobranchius shows positive selection in GSTK1 contains, in addition, one site with high probability of positive selection in the LCA of Nothobranchius (Glu167, blue in Fig. 2). The selected site in Nothobranchius, however, is structurally remote to the functionally relevant sites. In contrast, we found that in clownfish two of three sites that were predicted with high probability to be positively selected (≥ 95%, Phe60, Met63, red in Fig. 2) and one of three sites with lower probability (41%, His64, orange in Fig. 2) belong to the same α-helical stretch of amino acids that lines the substrate access channel, contribute to the dimer interface (Asn61, Tyr65, Asp69, green in Fig. 2) as well as to the substrate binding sites (Lys62, turquoise in Fig. 2), respectively [55]. The third site with a high probability to be positively selected is Glu88 (brown in Fig. 2). Glu88 is one of four amino acids at the entrance of the substrate access channel and situated in close proximity to Pro55, Pro56 and Pro87 (black in Fig. 2). The latter three are also part of the substrate access channel [55]. We found another site positively selected with a lower probability in close proximity to the dimer interface (Lys177, orange in Fig. 2). This positive selection at particular positions related to enzymatic function invites the speculation that it might have a bearing on the enzymatic activity of the clownfish GSTK1.

### Meta-analysis suggests that adaptation of mitochondrial biogenesis is a key player in evolution of lifespans

Recent observations of similar genes and pathways found to be affected by positive selection, both, in very long- and short-lived species led to hypotheses of antiparallel evolution acting on these entities [56, 57]. This means that functionally opposite selection pressures with regard to the tradeoff between fast growth and a long lifespan can result in adaptations of the same genes and pathways – in opposite functional directions.

This applies particularly for genes involved in mitochondrial biogenesis: Functions like “Mitochondrial large/small ribosomal subunit” (GO:0005762/ GO:0005763) and “Mitochondrial respiratory chain complex I” (GO:0005747) were found to be enriched for PSGs on ant branches that are associated with a 10- to 100-fold increase in lifespan [58, 59]. Similar genes, associated with mitochondrial functions, were found to be under positive selection in the bats *P. poliocephalus* and *M. lucifugus* that can reach lifespans of more than 20 and 30 years, respectively [60, 61]. On the other hand, also extreme reduction of lifespan on three killifish branches (*Nothobranchius* family) was associated with an enrichment of PSGs for a gene set that stands explicitly for mitochondrial biogenesis ([62], *p* < 10^− 6^).

We examined this hypothesis of lifespan-associated, antiparallel evolution of mitochondrial biogenesis genes by testing whether the PSGs identified on the clownfish ancestor are enriched in the same gene set that was used in the killifish study. Furthermore, we reanalyzed in the same regard PSGs that were identified across mole-rat branches on which lifespans were remarkably prolonged. In both cases – clownfish ancestor as well as long-lived mole-rats – we found again an enrichment of PSGs for genes involved in mitochondrial biogenesis (*p* = 0.007 and *p* = 0.097, respectively, Additional file 1: Table S7).

We further tested the hypothesis by using Fisher’s method to combine enrichment *p*-values across the results of the mentioned positive selection analyses in short-lived killifishes, long-lived mole-rats, and long-lived clownfishes. In this meta-analysis, 34 genes exhibited a signature of positive selection (FDR < 0.1) across species (Additional file 1: Tables S4, S5 and S6). Among the genes involved in mitochondrial biogenesis were *TFB2M* and *MTERF*, that are necessary for mitochondrial transcription, *FASTKD5* and *FASTKD2* whose gene products are required for the biogenesis of mitochondrial ribosomes, [63], as well as *RARS2* coding for a mitochondrial tRNA-synthetase. Again, the 34 genes that were significant in the meta-analysis across three different analysis associated with lifespan-changes were enriched mitochondrial biogenesis gene set (*p* = 1.05*10^− 5^, Additional file 1: Table S7).

Among the other 15 PSGs genes showing evidence for positive selection, both, in the clownfish LCA and in meta-analysis were, e.g., *LAMP2* and *CD63* (also called *LAMP3*) which code for major protein components of the lysosomal membrane [64, 65]. In addition, *CD63* gene expression was shown to predict the malignancy grade of many different tumor types [66–70] and the artificial prevention of the decrease of *LAMP2* gene expression during aging in mice results in considerably reduced cell damage, as well as in liver functions in old mice that are indistinguishable from those in young mice [71]. Finally, another interesting example that was identified as significant, both, in the clownfish LCA and in the meta-analysis, is *GSTK1* encoding a glutathione-S-transferase that localizes to the peroxisome. GSTK1 was shown to be associated with diabetes type 2 which is another major aging related disease [72, 73].

As a negative control, we searched for PSGs on evolutionary branches that were not associated with drastic changes of lifespan. For this, we used the closely-related sister-taxons of the three above mentioned species: *C. viridis* as sister-taxon of the clownfish-ancestor, *C. porcellus* (guinea pig) for the mole-rats and *Aphysemion striatum* for killifishes. 188, 124 and 27 PSGs were identified, respectively (FDR < 0.05, Additional file 1: Tables S8, S9 and S10). On none of these branches, however, an enrichment of PSGs against the mitochondrial biogenesis gene set or any related gene ontology category was found. Neither were the PSGs that were identified as significant (FDR < 0.1) in the meta-analysis across the three branches enriched for the mitochondrial biogenesis gene set (Additional file 1: Table S11) (Table 3).

**Table 3:** Positively selected genes associated with mitochondrial biogenesis identified in a meta-analysis across three evolutionary clades with exceptional short or long lifespans

Gene symbol	p-value				FDR^d^
	Clownfish LCA^a^	*Nothobranchius* ^b^	Mole-rat^b^	Combined^c^	
MTERF1	7.34E-03	1.25E-03	2.57E-02	3.12E-05	4.01E-04
RARS2	1.82E-03	1.18E-02	NA^e^	2.52E-04	2.43E-03
MRPL30	2.55E-02	1.00E+ 00	9.59E-03	2.28E-03	7.98E-03
FASTKD2	1.77E-04	1.56E-01	1.00E+ 00	3.16E-04	2.43E-03
FASTKD5	NA^e^	1.39E-03	8.25E-01	8.89E-03	2.44E-02
TFB2M	NA^e^	4.34E-04	6.91E-01	2.73E-03	9.15E-03
NDUFA9	NA^e^	8.31E-02	6.90E-03	4.85E-03	1.56E-02

^a^ LCA – last common ancestors

^b^ These *p*-values resulted from meta-analysis using Fisher’s method of 3 ancestral Nothobranchius and 11 examined mole-rat branches on which lifespan changed considerably

^c^ This p-value results from a meta-analysis of the three p-values in the left columns using Fisher’s method

^d^ FDR – false discovery rate (adjusted p-value for multiple testing)

^e^ NA – no p-value calculated since the gene could not be tested in the respective context

## Discussion

We have corroborated the evidence for exceptional longevity of clownfishes in captivity. The species *A. ocellaris* is bred in captivity and commercially available in large numbers and it could become the first broadly-available laboratory model for extended lifespan and exceptional longevity.

Analysis of positive selection has shown evolutionary convergence, both, with the exceptionally short-lived genus *Nothobranchius* and with exceptionally-long lived mole rats.

In particular, clownfishes and mole rats both show positive selection in two key proteins of the lysosome: LAMP2 and CD63. These results are consistent with the conserved up-regulation across tissues and species of genes coding for lysosomal proteins [74, 75], the widespread accumulation of lysosomal aggregates observed during aging [76] and earlier findings that associated selection on lysosomal genes with evolution of mammalian longevity [9]. This suggests that lysosomal function is of key importance for evolution of exceptional longevity. Another interesting example of convergent evolution is GSTK1, which is positively selected in both the exceptionally-long and exceptionally-short lived fish clades. GSTK1 is involved in glutathione metabolism. Since detailed structures of this protein are available [54, 55], homology modelling was possible and it strongly suggests that positive selection targeted positions that are involved in the enzymatic function of the encoded protein. This speculation would have to be tested experimentally.

Finally, prominent signs of convergence were observed for genes and pathways related to biogenesis of mitochondrially-encoded proteins with the remarkable observation that MTERF is under positive selection in all three taxa. These findings parallel experimental evidence obtained in laboratory species where reduced expression of mitochondrially-encoded proteins prolongs lifespan [52, 53] and point to the key importance of mito-nuclear balance in the evolution of animal longevity. Further, the observation that the same pathway is under positive selection both in exceptionally short- and long-lived species indicate that the same genetic architecture underlies both evolution of longevity and evolution of compressed lifespan.

## Conclusions

The clownfish represents the first vertebrate model of exceptional longevity that can be easily cultivated in captivity.

Analysis of positive selection on protein-coding genes reveals convergence on lysosomal protein with long-lived mole rats and convergence on mito-nuclear balance genes with both mole-rats and the exceptionally short-lived annual killifishes.

These data indicate that the same genetic pathways were repeatedly recruited in response to evolution of lifespan in either direction.

## Methods

### Clownfish lifespan estimation

The determination of the clownfish lifespan was performed through the distribution of an internet-based questionnaire to zoos and aquariums worldwide, requesting information on clownfish demographic details: (1) the various clownfish species maintained in captivity, (2) the number of individuals for each species, (3) if each individual is captive bred or not, (4) the year of acquisition and, if not still alive, death, and (5) the sex of each individual, if determined. The questionnaire was circulated in 2016 to international associations and organizations of zoos and public aquariums such as the European Association of Zoos and Aquaria (EAZA), the Association of Zoos and Aquariums (AZA), the European Union of Aquarium Curators (EUAC) and the World Association of Zoos and Aquariums (WAZA). Responses to our questionnaire were received from 5 zoos and aquariums as well as two private entities resulting in lifespan data for 114 individuals (see Additional file 1: Table S1 and Acknowledgments).

### Experimental fish and sampling

Sub adult *Amphiprion percula* (total length, 45.2 ± 1.2 mm; Wt, 1.6 ± 0.1 g, *n* = 12), *Amphiprion clarkii* (total length, 46.4 ± 5.1 mm; Wt, 2.3 ± 0.9 g, n = 12) and *Chromis viridis* (total length, 43.0 ± 1.6 mm; Wt, 1.3 ± 0.1 g, n = 12), were used. Animals were acquired from local dealers and subjected to acclimation during one month in the facilities of the Marine Aquarium at the University of Murcia (Spain). Fishes were kept in groups under exactly the same conditions (temperature, 27 ± 1 °C; salinity, 24 ± 1, pH, 8 ± 0.2; dissolved oxygen, 6.5 ± 0.2 mg/L) and fed ad libitum four times a day a standard low-fat diet to match their requirements (composed by Mysis shrimp, enriched *Artemia nauplii* and red plankton).

Fishes were euthanized by exposure to the anesthetic benzocaine hydrochloride (400 mg l^− 1^) for 10 min following the cessation of gill movement. Brains, livers and samples of skeletal muscle were collected for analyses. For each species, three whole brains were frozen in liiquid nitrogen and stored at − 80 °C prior to molecular determinations.

The animal procedures were approved by responsible authorities (A13160603, from the Consejeria de Agua, Agricultura, Ganaderia y Pesca, Comunidad Autonoma de la Region de Murcia, Spain).

### Coding sequence data

Our analysis comprised five clownfish species (*A. ocellaris*, *A. clarkii*, *A. bicinctus*, *A. percula*, *A. sebae*), *C. viridis* representing the non-symbiotic sister-taxon of the *Amphiprion* genus and nine more distantly related outgroup species (*Stegastes partitus*, *Pundamilia nyererei*, *Maylandia zebra*, *Oryzias latipes*, *Xiphophorus maculatus*, *Poecilia formosa*, *Fundulus heteroclitus*, *Nothobranchius furzeri*, *Aphyosemion striatum*). mRNA sequences of the outgroups were obtained from RefSeq along with their coding sequence annotation (Additional file 1: Table S12). For *A. ocellaris*, *A. bicinctus* and *A. sebae*, we downloaded read data from the short read archive (Bio projects PRJNA374650, PRJNA261388 and PRJNA285007, respectively). For *A. clarkii*, *A. percula* and *C. viridis*, we performed novel RNA-seq using samples from three individuals per species, the Qiagen RNeasy Mini Kit for purification, an Illumina HiSeq 2500 sequencing device (rapid run mode) and a paired end sequencing strategy with a read length of 150 base pairs. The number of resulting read pairs per sample was between 26 and 47 million reads (see Additional file 1: Table S13 for more details). The reads of the clownfishes and *C. viridis* were preprocessed using SeqPrep with minimum adapter length of five as well as a demanded minimum read length of 50. De novo transcriptome assemblies for these species were performed using FRAMA with *Stegastes partitus* as reference species [77]. For the clownfishes and *C. viridis* the longest isoform was chosen to represent the gene. For the outgroups, in cases in which multiple isoforms per gene were annotated based on the reference, all of them were used in subsequent analyses. The assembly completeness of all examined species was estimated using BUSCO [78], was 90–100% (Additional file 1: Table S12).

### Identification of positively selected genes

To scan on a genome-wide scale for genes under positive selection, we fed the coding sequences of the described species set into the PosiGene pipeline [79]. *Stegastes partitus* was used as PosiGene’s anchor species. Orthology was determined by PosiGene via best bidirectional BLAST searches [80, 81] against *Stegastes partitus*. Within the analysis of positive selection PosiGene determined among others alignments of the orthologous genes using PRANK [82], reconstructed a phylogenetic tree of the examined species with DNAML from the PHYLIP package [83] and applied the branch-site test of positive selection [84] by using PAML [85]. The branches of the last common ancestor of the clownfishes and *C. viridis* were tested separately for genes under positive selection (Additional file 1: Table S2). FDR < 0.05 was used as threshold for significance. Assembled sequence data, visualizations of alignments and positively selected sites were made available for download (see Additional file 2).

Furthermore, PSGs for *A. striatum* and *C. porcellus* were determined using the same sequences, species and PosiGene settings as described in the original killifish and mole-rat study, respectively [8, 23].

### Gene ontologies

We determined enrichments for GO categories using Fisher’s exact test based on the R package GOstats (Table 2 and Additional file 1: Table S3). The resulting *p*-values were corrected using the Benjamini-Hochberg method [86]. We used throughout the manuscript 0.1 as significance threshold. Applying implementations of Benjamini-Hochberg correction to raw *p*-values often result in same FDR values for multiple elements despite that they have different p-values, e.g. in row 3 to row 16 of Table 2 and Additional file 1: Table S3. The reason for this behavior is that most implementations of this method want to ensure that the sortation of elements by FDR is the same as by p-value. Therefore, they calculate the respective FDR values for the p-values p_1_ ≤ p_2_ ≤ … ≤ p_n_ starting with FDR_n_ and then set FDR_i_:= FDR_i + 1_, for 1 ≤ i < n in case that otherwise (according to the formula of the FDR) FDR_i_ would be greater than FDR_i + 1_. By chance this happens, e.g., in the case of Table 2/ Additional file 1: Table S3 from *FDR*_3_ to *FDR*_16_ several times in a row.

Enrichment for mitochondrial biogenesis genes was tested using Fisher’s exact test (for the clownfish LCA, *C. viridis*, *A. striatum* and *C. porcellus*) and the union set of the genes in the following five mitochondrial related GO terms: GO:0000959, 0032543, 0045333, 0033108, 0070584 (Additional file 1: Table S5). The same GO terms were used in our previous study [23] to test for enrichment. In the case of the re-analyzation of the mole-rat study [8] with regard to mitochondrial biogenesis, Fisher’s exact test was not applicable since enrichment for multiple branches (in total 11) with different background gene sets had to be tested at the same time. Therefore, a resampling approach was used for p-value calculation analogous to the one that was used to test the three killifish branches at the same time for enrichment of the mitochondrial biogenesis gene set [23].

The relationship between aging and the results from the positive selection analysis on the clownfish LCA was tested based on a list of 41 gene ontology functions whose gene expressions were found to be associated with maximum lifespan residual in a study across 33 mammals [12]. This means, that this study found the expression of the genes in those gene ontologies to be correlated or anti-correlated with the difference between observed maximum lifespan and expected maximum lifespan based on the weight of the respective species. Out of these 41 functions, 11 were tested during our enrichment analysis. We used Fisher’s method to compute the *p*-values of these 11 gene ontology processes from our enrichment analysis into a test statistic. The latter was tested against a null distribution that was empirically estimated from 100,000 random drawings of the p-values from enrichment analysis.

### Meta analysis

To identify genes that show signs of positive selection across multiple evolutionary branches on which the lifespan was altered considerably, we combined p-values from this study with those of two previous studies using Fisher’s method [87] (Additional file 1: Table S4, S5 and S6). In all three studies, PosiGene was used to determine p-values. The first study searched for genes under positive selection on 11 rodent branches in which the lifespan was presumably extended – most of them in the clade of the African mole-rat family that covers the longest-lived known rodents [61]. The second study examined three branches of the *Nothobranchius* genus on which lifespan was presumably reduced [23] – the genus covers the shortest-lived vertebrate species that can be held in captivity [88]. As significance threshold for meta-analysis p-values, 0.1 was chosen throughout the manuscript.

### Protein homology modeling

Homology modelling of the clownfish GSTK1 was carried out with SWISS–MODEL (http://swissmodel.expasy.org; [89, 90] using the crystal structures of the dimeric apoform of the human mitochondrial GSTK1 (PDB 3rpp; [54]) and the substrate bound dimer of the rat enzyme PDB 1r4w; [55]). No further optimization was applied to the resulting models. Visualization, superimposition of the respective crystal structures and the models as well as rendering was carried out using CHIMERA [91]. Figure 2c shows one subunit of the modelled dimer of the Clownfish GSTK1 model. Additional file 3: Figure S1 provides an overview over both subunits.

## Additional files

Additional file 1: **Table S1**. Clownfish lifespan questionnaire results. **Table S2**. PosiGene results for positively selected genes on the phylogenetic branch representing the last common ancestor of the clownfishes (genus *Amphiprion*). **Table S3**. Enrichment test results of the biological gene ontology processes enriched for positively selected genes. **Table S4**. Meta analysis using Fisher’s method of positive selection across three analyses of phylogenetic branches on which lifespan changed considerably. **Table S5**. Meta analysis using Fisher’s method of positive selection across three phylogenetic branches of the Nothobranchius genus on which the lifespan was reduced considerably. **Table S6**. Meta analysis using Fisher’s method of positive selection across eleven phylogenetic rodent branches on which the lifespan was reduced considerably. **Table S7**. Genes that were regarded as mitochondrial biogenesis related from five gene ontology terms. **Table S8**. PosiGene results for positively selected genes on the phylogenetic branch representing *Chromis viridis*. **Table S9**. PosiGene results for positively selected genes on the phylogenetic branch representing *Cavia porcellus*. **Table S10**. PosiGene results for positively selected genes on the phylogenetic branch representing Aphysemion striatum. **Table S11**. Meta analysis using Fisher’s method of positive selection across three analyses of phylogenetic branches on which the lifespan did not change considerably. **Table S12**. Assembly and sequence statistics. **Table S13**. Samples that were sequenced to create genome/transcriptome assemblies. (XLSX 6532 kb)

Additional file 2: Supplement data. (DOCX 12 kb)

Additional file 3: **Figure S1.** Homology modelling of Clownfish GSTK1. Ribbon representation of the model dimer for the clownfish enzyme as derived from SWISS-MODEL in grey, superimposed onto the dimeric structure of the substrate bound rat GSTK1 (PDB 1r4w; [55]) used as template in light green. The pairwise root mean square deviation for the Cα positions between the model and 1r4w amounts to 0.52 Å as determined with the CHIMERA Matchmaker tool. The GSH substrate in the rat enzyme structure is depicted in light purple. (PNG 1250 kb)

## Abbreviations

FDR: False discovery rate
GO: Gene ontology
GSH: Glutathione
GSTK1: Glutathione S-transferase kappa 1
LCA: Last common ancestor
MTERF1: Mitochondrial transcription termination factor 1
PSG: Positively selected gene
