# Supplementary material for: Analysis of the coding sequences of clownfish reveals molecular convergence in the evolution of lifespan
Source: BMC Evol Biol. 2019 Apr 11;19:89. doi: 10.1186/s12862-019-1409-0 (PMC6460853; doi:10.1186/s12862-019-1409-0)
Supplement: Supplementary file 2 — Supplement data. (DOCX 12 kb) [file 12862_2019_1409_MOESM2_ESM.docx]

Additional file 2: Supplement data

available at:

https://doi.org/10.1101/380709

OR

ftp://genome.leibniz-fli.de/pub/user/arne.sahm/clownfish/supplement_data.tar.gz

The package contains assembled sequence data, visualizations of alignments and positively selected sites for all genes that were analyzed in this article.
