# Supplementary figures and images for: Analysis of the coding sequences of clownfish reveals molecular convergence in the evolution of lifespan
Source: BMC Evol Biol. 2019 Apr 11;19:89. doi: 10.1186/s12862-019-1409-0 (PMC6460853; doi:10.1186/s12862-019-1409-0)

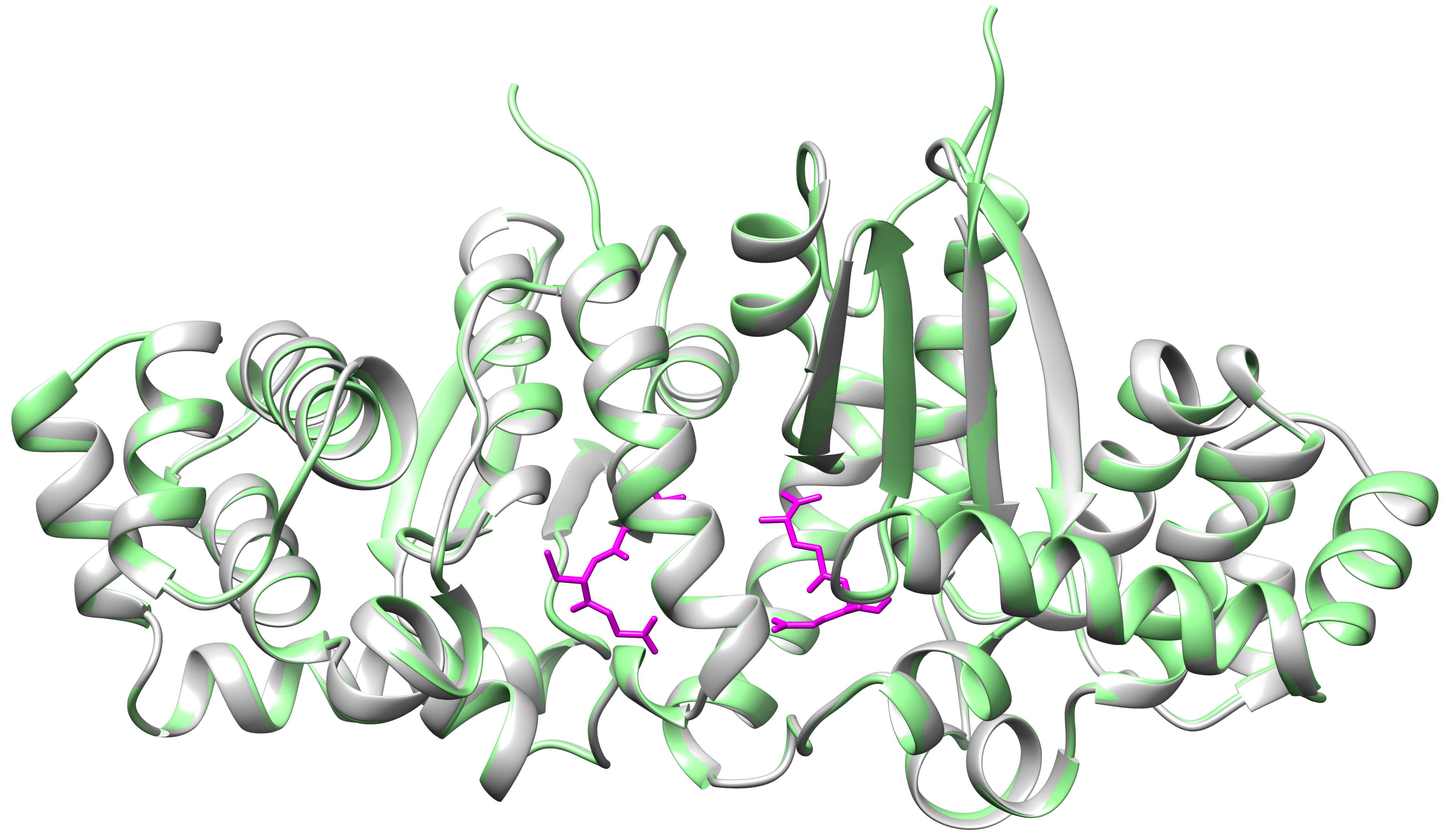

Supplement: Supplementary file 3 — Figure S1. Homology modelling of Clownfish GSTK1. Ribbon representation of the model dimer for the clownfish enzyme as derived from SWISS-MODEL in grey, superimposed onto the dimeric structure of the substrate bound rat GSTK1 (PDB 1r4w; [55]) used as template in light green. The pairwise root mean square deviation for the Cα positions between the model and 1r4w amounts to 0.52 Å as determined with the CHIMERA Matchmaker tool. The GSH substrate in the rat enzyme structure is depicted in light purple. (PNG 1250 kb) [file 12862_2019_1409_MOESM3_ESM.png]
